# High‐dose wogonin exacerbates DSS‐induced colitis by up‐regulating effector T cell function and inhibiting Treg cell

**DOI:** 10.1111/jcmm.12964

**Published:** 2016-09-19

**Authors:** Weiming Xiao, Min Yin, Keyan Wu, Guotao Lu, Bin Deng, Yu Zhang, Li Qian, Xiaoqing Jia, Yanbing Ding, Weijuan Gong

**Affiliations:** ^1^ Department of GastroenterologyAffiliated hospitalYangzhou UniversityYangzhouChina; ^2^ Jiangsu Key Laboratory of Integrated Traditional Chinese and Western Medicine for Prevention and Treatment of Senile DiseasesYangzhouChina; ^3^ Department of ImmunologySchool of MedicineYangzhou UniversityYangzhouChina; ^4^ Jiangsu Key Laboratory of ZoonosisYangzhouChina; ^5^ Jiangsu Co‐innovation Center for Prevention and Control of Important Animal Infectious Diseases and ZoonosesYangzhouChina; ^6^ Key Research Laboratory of Theory and Treatment on Toxicity of Stomach CancerState Administration of Traditional Chinese MedicineYangzhouChina

**Keywords:** wogonin, exacerbate, colitis, effector T cell, regulatory T cell

## Abstract

Wogonin exerts anti‐tumour activities *via* multiple mechanisms. We have identified that high‐dose wogonin (50 or 100 mg/kg) could inhibit the growth of transplanted tumours by directly inducing tumour apoptosis and promoting DC, T and NK cell recruitment into tumour tissues to enhance immune surveillance. However, wogonin (20–50 μM) *ex vivo* prevents inflammation by inhibiting NF‐κB and Erk signalling of macrophages and epithelial cells. It is elusive whether high‐dose wogonin promotes or prevents inflammation. To investigate the effects of high‐dose wogonin on murine colitis induced by dextran sodium sulphate (DSS), mice were co‐treated with DSS and various doses of wogonin. Intraperitoneal administration of wogonin (100 mg/kg) exacerbated DSS‐induced murine colitis. More CD4^+^ CD44^+^ and CD8^+^ CD44^+^ cells were located in the inflamed colons in the wogonin (100 mg/kg) treatment group than in the other groups. Frequencies of CD4^+^ CD25^+^ CD127^−^ and CD4^+^ CD25^+^ Foxp3^+^ cells in the colons and spleen respectively, were reduced by wogonin treatment. *Ex vivo* stimulations with high‐dose wogonin (50–100 μg/ml equivalent to 176–352 μM) could synergize with IL‐2 to promote the functions of CD4^+^ and CD8^+^ cells. However, regulatory T cell induction was inhibited. Wogonin stimulated the activation of NF‐κB and Erk but down‐regulated STAT3 phosphorylation in the CD4^+^ T cells. Wogonin down‐regulated Erk and STAT3‐Y705 phosphorylation in the regulatory T cells but promoted NF‐κB and STAT3‐S727 activation. Our study demonstrated that high‐dose wogonin treatments would enhance immune activity by stimulating the effector T cells and by down‐regulating regulatory T cells.

## Introduction

Wogonin as a natural flavonoid from *Scutellaria baicalensis* Georgi (Lamiaceae) exhibits anti‐tumour activity 1, 2, 3. This compound at dosages of 50–200 μM *ex vivo* kills tumours by up‐regulating intracellular reactive oxygen species 4, arresting cell cycle, inducing apoptosis 5, 6, reversing drug resistance 7 and inhibiting angiogenesis 8, 9. Wogonin down‐regulates the PI3K‐Akt pathway, thereby suppressing LPS‐ or H_2_O_2_‐induced angiogenesis 10. NF‐κB 11 and Nrf2 12 signalling pathways are also involved in wogonin‐mediated inhibition of inflammation‐associated colorectal carcinogenesis. Wogonin induces Erk phosphorylation 13 and activates p38MAPK 14 to trigger apoptosis of tumour cells. Wogonin also up‐regulates the expression of p21, p27 and p53 to induce tumour cell cycle arrest at the G1/S phase 15.

Using Wogonin at 20–50 μM also displays anti‐inflammatory activity by regulating the macrophage function 16, 17. The flavonoid (30 μM) could attenuate endotoxin‐induced prostaglandin E2 and nitric oxide production *via* the Src‐Erk1/2‐NF‐κB pathway in BV‐2 microglial cells 18. Wogonin (40 mg/kg) reduced the activation of TLR4/NF‐κB signalling after experimental traumatic brain injury 19. Wogonin (30 mg/kg) also prevented lipopolysaccharide‐induced acute lung injury and inflammation in mice *via* peroxisome proliferator‐activated receptor gamma‐mediated attenuation of NF‐κB pathway 20. Moreover, wogonin (<10 μM) inhibited the up‐regulation of receptor activator of NF‐κB expression and down‐regulation of osteoprotegerin expression by LPS in osteoblasts 21. However, wogonin is a relatively safe drug, because the LD (50) of wogonin administered by the intravenous injection in mice was 286.15 mg/kg and the 95% confidence limit was 278.27–295.26 mg/kg 22.

The effects of wogonin on T cell function under different micro‐environments remain ambiguous. Mid‐dose (20 mg/kg) wogonin treatment significantly inhibited chronic colitis induced by dextran sodium sulphate (DSS) within 2 weeks through the down‐regulation of Th2‐associated cytokine, particularly IL‐4 and IL‐10 secretion 23. Wogonin also down‐regulates OVA‐induced Th2 immune responses, particularly IgE and IL‐5 prediction 24. However, IFN‐γ and IL‐2 production of T cells co‐stimulated by concanavalin A and wogonin has been shown to be significantly enhanced 23. Wogonin also inhibits tumour‐mediated induction of Treg cells by inhibiting TGF‐β1 activity 25. We found that wogonin administered at 50 and 100 mg/kg inhibited tumour growth and promoted the recruitment of DC, T, and NK cells in the tumour tissues in the xenograft tumour model of mice 26. In the current study, the effect of high‐dose wogonin on the onset of DSS‐induced acute colitis was determined. Moreover, the effects of high‐dose wogonin on the function of the effector T and regulatory T cell were examined.

## Materials and methods

### Animals and cell lines

C57BL/6 mice, aged 6–8 weeks, were purchased from the Comparative Medicine Centre of Yangzhou University (Yangzhou, China). The mouse gastric cancer cell line (MFC) was from Shanghai cell bank of Chinese Academy of Sciences. MFC cells were adherent and subcultured every 3 days. The murine colon cancer cell line (MC‐38) was kindly gifted by Dr. Hursting (University of Texas‐Austin). Both cells were cultured in RPMI 1640 (Gibco, Grand Island, NY, USA) supplemented with 10% foetal bovine serum (FBS; Gibco), 100 U/ml penicillin, and 100 μg/ml streptomycin sulphate (Beyotime, Jiangsu, China). For storage, cell lines were suspended in complete growth medium supplemented with 5% (v/v) DMSO and located in liquid nitrogen vapour phase.

### Drugs and reagents

Wogonin (purity >98%) purchased from Nanjing Zelang Medical Technology (Nanjing, Jiangsu, China) was dissolved in 1 M NaOH as a stock solution, stored at −20°C, and freshly diluted with RPMI 1640 medium to the final concentration. The working solution of NaOH was less than 0.1 μM. DSS (molecular weight: 36,000–50,000) was obtained from MP Biomedical (Solon, Ohio, USA). Lymphoprep was obtained from Axis‐shield (Oslo, Norway). Collagenase IV, Dnase I and Percoll were purchased from BIOSHARP (Hefei, Anhui, China). Dispase II was obtained from Roche (Basel, Switzerland).

### Antibodies for flow cytometry and Western blot analysis

The antibodies for flow cytometry were obtained from Biolegend (San Diego, CA, USA) or eBioscience (San Diego, CA, USA). We used antibodies against mouse CD4 (GK1.5), CD8 (53–6.7), CD69 (H1.2F3), CD44 (IM7), INF‐γ (XMG1.2), CD4 (RM4‐5), CD25 (PC61.5), Foxp3 (NRRF‐30) and CD127 (SB/199). Cell apoptosis 7‐amino‐actinomycin D (7‐AAD) detection kit was purchased from KeyGEN (Jiangsu, China). Antibodies against STAT3 (79D7), phospho‐Stat3(Tyr705) (D3A7), phospho‐Stat3 (Ser727) (07‐703), NF‐κB p65(ABE136), phospho‐NF‐κB p65 (Ser536), p44/42 MAPK(Erk1/2) (137F5), phospho‐p44/42 MAPK(Erk1/2) (Thr202/Tyr204) (D13.14.4E) and GAPDH(D16H11) for Western blot analysis were from Cell Signaling (Boston, MA, USA) or Merck & Millipore (Billerica, MA, USA).

### DSS‐induced colitis

Acute colitis was induced by administering DSS (2.5%) in drinking water from days 1 to 7. The mice were randomly divided into solvent control, DSS and solvent‐treated, DSS and wogonin (20 mg/kg)‐treated, DSS and wogonin (50 mg/kg)‐treated, and DSS and wogonin (100 mg/kg)‐treated groups. Wogonin or solvent was intraperitoneally injected daily from days 1 to 7. All mice were weighed every day. The mice were killed on day 8, and the spleens and intestinal tissues were removed for *ex vivo* analysis. All experimental protocols were approved by the Institutional Animal Care and Use Committee of Yangzhou University.

### Isolation and detection of colonic mononuclear cells of mice

The intestinal tissues of mice were washed repeatedly with phosphate‐buffered saline (PBS) using 5‐ml syringe until the intestines became translucent. The tissue was opened longitudinally, cut into 5‐mm pieces, and incubated in 5 mM EDTA and 1 mM DTT in calcium‐free and magnesium‐free Hank's balanced salt solution for 20 min. at 37°C. The tissue was then centrifuged, and the supernatant was collected. The residual tissue was incubated again in a digestion solution containing 0.5 mg/ml of type IV collagenase or Dnase I, as well as 3 mg/ml of Dispase II for 30 min. at 37°C. The suspension combined with the previous supernatant was centrifuged, and the mononuclear cells were isolated by centrifugation in 35% Percoll solution 27. The cell suspensions were incubated with 7‐AAD to exclude dead cells stained with mAbs against CD4, CD25 and CD127. The cells were analysed by flow cytometry.

### Real‐time PCR

Total RNA was isolated from murine CD8^+^ T or CD4^+^ T cells after wogonin treatments by the TRIzol reagent (Life Technologies, Carlsbad, CA, USA). Reverse transcription was conducted for 15 min. at 42°C with 1 μg of total RNA using a QuantiTect Reverse Transcription Kit (QIAGEN GmbH, Hilden, Germany). Quantitative RT‐PCR reaction was monitored using the ABI 7500 (PE applied Biosystems, Carlsbad, CA, USA) and the results were analysed with the accompanying software. The SYBR Green PCR Kit (QIAGEN GmbH) was used for detection of mouse IFN‐γ and GAPDH. The primer pairs are shown below: 5′‐CTGTTTCTG GCTGTTACTGC‐3′ and 5′‐TGCTGATGGCCTGATTGT‐3′ (IFN‐γ);5′‐CAAAATGGTGAAGGTCGGTGTG‐3′ and 5′‐TGATGTTAGTGGGGTCT CGCTC‐3′ (GAPDH).The relative fluorescence of IFN‐γ *versus* GAPDH was analysed by densitometry. Relative RNA expression was calculated by the 2^−▵▵Ct^ method after normalizing expression levels of IFN‐γ mRNA to GAPDH mRNA.

### Magnetic cell sorting

Splenic CD4^+^ CD25^−^ T cells of all groups of mice were obtained from a double‐negative magnetic cell sorting (MACS). The Miltenyi CD4 cell isolation kit was used to label splenocytes with 100 μl of the biotinylated cocktail of antibodies (against CD8a, CD11b, CD11c, CD19, B220, CD49b, CD105, MHC‐class II and Ter‐119) specific for CD4^−^ cells diluted in 400 μl of RPMI 10% FBS for 15 min. at 4°C. Then, 200 μl of the anti‐biotin antibody conjugated with microbeads was added, and the cells were incubated for 10 min. at 4°C. After washing, the cells were applied on an LD separation column (Miltenyi, Teterow, Germany). CD4^−^ cells remained in the column because of the magnetic beads attached to their surface. By contrast, CD4^+^ cells flowed through the column and could be collected in a tube. The untouched CD4^+^ cells were further indirectly labelled with an anti‐CD25‐PE and anti‐PE conjugated with microbeads. Cells were flowed on an LS column, and CD4^+^ CD25^−^ cells were collected. The purity of CD4^+^ CD25^−^ T cells was more than 90% as identified by flow cytometry.

### *In vitro* induction of CD4^+^ CD25^+^ regulatory T cells

A 24‐well plate was coated with 10 μg/ml anti‐CD3 antibody in PBS at 37°C for 2 hrs and then washed once with PBS before cell plating. CD4^+^ CD25^−^ T cells obtained from MACS were resuspended in serum‐free medium in anti‐CD3‐precoated wells without antibiotics. Sorted cells were stimulated with anti‐CD28 antibody (2 μg/ml), IL‐2 (20 U/ml) and TGF‐β (5 ng/ml) for 5 days [28, 29]. On day 6, cells were treated with various doses of wogonin for 24 hrs, and solvent was used as a negative control. Finally, the cells were collected and analysed by flow cytometry to detect the amount of CD4^+^ CD25^+^ Foxp3^+^ T cells.

### Western blot analysis

After CD4^+^ T cells, or CD4^+^ CD25^+^ regulatory T cells which were induced by IL‐2, CD28 antibody and TGF‐β above, were treated with different doses of wogonin, all cells were harvested, washed once in ice‐cold PBS and gently lysed in ice‐cold lysis buffer respectively. Proteins were transferred to polyvinylidene fluoride (PVDF) membranes and blocked successively in 5% bovine serum albumin in TBST for 1 hr. The membranes were then incubated overnight at 4°C with antibodies, including Stat3 (1:2000), phospho‐Stat3 (Tyr705) (1:2000), anti‐phospho‐STAT3 (Ser727) (1:1000), anti‐NF‐κB p65 (1:2500), phospho‐NF‐κB p65 (1:1000), p44/42 MAPK (Erk1/2) (1:1000), phospho‐p44/42 MAPK (Erk1/2) (1:2000) and GAPDH (1:1000). GADPH was used as a negative control. PVDF membranes were then incubated with horseradish peroxidase‐labelled IgG (1:2500) at room temperature for 1 hr. Membranes were washed extensively after each incubation, and the immunoreactive bands were detected using ECL‐detecting reagents.

### Statistical analysis

Differences between groups were analysed using an unpaired, two‐tailed Student's *t*‐test. Data were evaluated by one‐way ANOVA followed by Dunnett's test between control and multiple dose groups. Significance of differences was indicated when *P < 0.05, **P < 0.01, and ***P < 0.001.

## Results

### Wogonin exacerbates DSS‐induced acute colitis of mice in association with T cell activation

**Figure 1:**
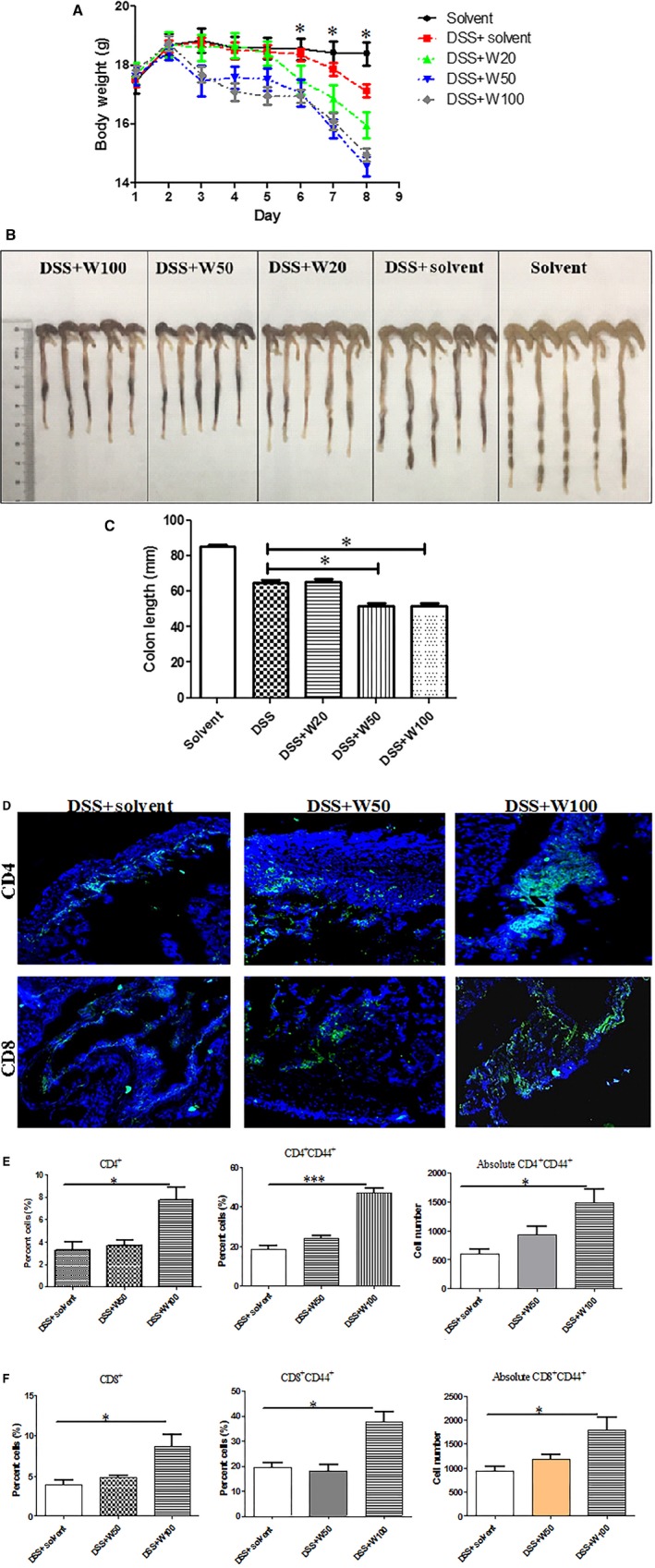
Wogonin exacerbates DSS‐induced colitis in mice associated with T cell infiltration. (**A**) Bodyweights of mice with DSS‐induced colitis treated by different wogonin dosages. *, represents comparison between DSS combined with 50 mg/kg or 100 mg/kg wognonin and DSS alone. (**B**) Morphological changes in the murine colons by wogonin treatments. (**C**) Variations of colon length of mice by wogonin treatments. **(D)** Infiltration of CD4^+^ T and CD8^+^ T cells of colons were detected by immunofluorescent assay. Photos were magnified by 200×. Green: CD4 or CD8 antibody; Blue: DAPI. Mononuclear colonic cells were separated and detected based on the total cell numbers and frequencies of **(E)** CD4^+^ and CD4^+^ CD44^+^ cells, and **(F)** CD8^+^ and CD8^+^ CD44^+^ cells. *, *P* < 0.05. All experiments were repeated five times. DSS, dextran sodium sulphate.

Treatment with 50 or 100 mg/kg wogonin accelerated the loss of bodyweight (Fig. 1A) and had the shortened colon length (Fig. 1B, C) of mice induced by DSS, particularly at 100 mg/kg. Treatment with DSS and 20 mg/kg wogonin did not show significant changes of weight and colon length compared with DSS alone. Intestinal bleeding and stenosis of mice in wogonin treatment groups (50 or 100 mg/kg) were more severe than those treated by solvent or DSS/solvent (Fig. 1B). Immunohistological analysis showed that more CD4^+^ and CD8^+^ T lymphocytes infiltrated the intestines of mice treated by 100 mg/kg wogonin (Fig. 1D), which indicated severe inflammation in the colons. Normal mice were also treated by wogonin (50 or 100 mg/kg) alone. There were not significant changes of bodyweight, colon length and histology of all groups of mice (Figure S1).

Intestinal mononuclear cells were isolated and analysed on CD4^+^ and CD8^+^ T cells by flow cytometry. Percentages of viable cells were more than 90% confirmed by 7‐AAD staining. The frequencies of colonic CD4^+^ cells and CD4^+^ CD44^+^ T cells were higher in mice treated with 100 mg/kg wogonin than in the solvent group. Moreover, the absolute CD4^+^ CD44^+^ T cell numbers in the colons of mice treated at the dosage were the highest among three groups (Fig. 1E). Meanwhile, the frequencies of CD8^+^ cells and CD8^+^ CD44^+^ T cells in the colons of mice treated with 100 mg/kg wogonin were significantly enhanced. Absolute numbers of colonic CD8^+^ CD44^+^ T cells in mice treated with 50 mg/kg wogonin were higher than in the solvent and 50 mg/kg wogonin‐treated groups (Fig. 1F). The CD44 expression of T cells indicated the activation and migration capacity of T lymphocytes 30. Thus, wogonin exacerbated DSS‐induced colitis by recruiting activated CD4^+^ and CD8^+^ cells into the colons.

### Wogonin treatment inhibited regulatory T cells in mice with DSS‐induced colitis

**Figure 2:**
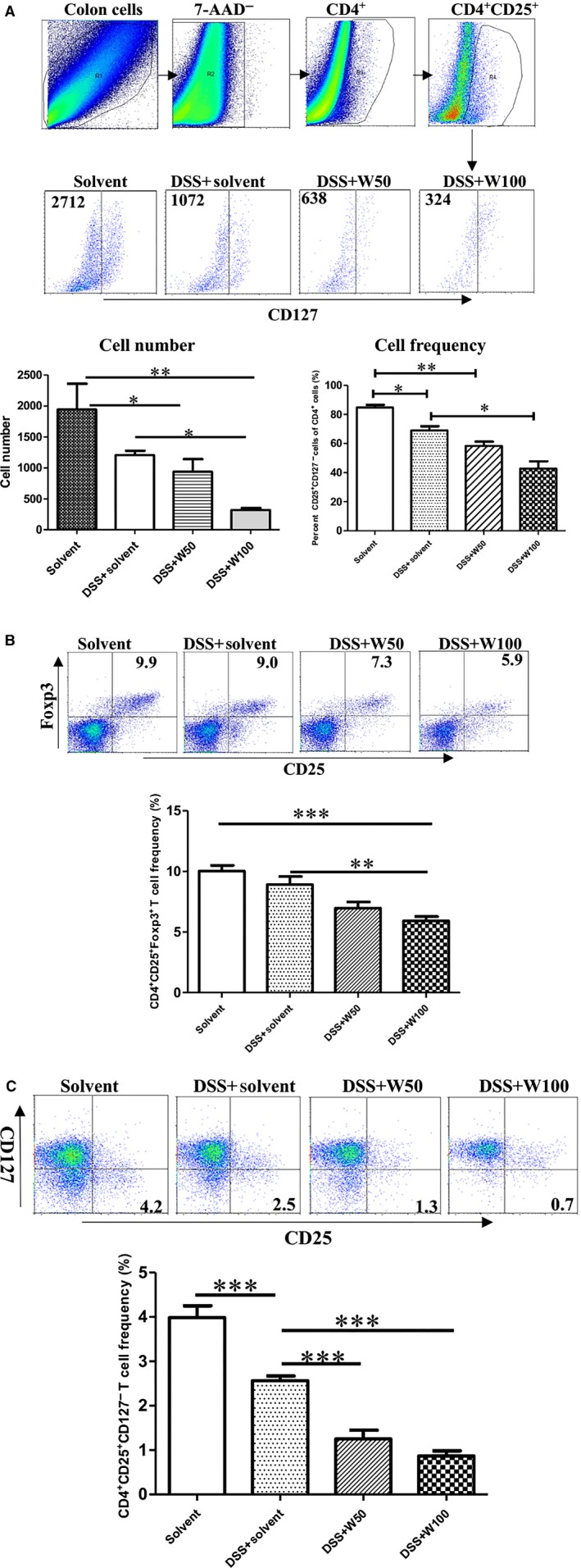
Lower frequency of regulatory T cells of colons treated by wogonin. **(A)** Numbers of CD4^+^ CD25^+^ CD127^−^ cells in the inflamed colons of mice stimulated by wogonin. Frequencies of **(B)** CD4^+^ CD25^+^ Foxp3^+^ and **(C)** CD4^+^ CD25^+^ CD127^−^ cells of spleens from mice after wogonin treatments were detected by flow cytometry. *, *P* < 0.05; **, *P* < 0.01; and ***, *P* < 0.001. The experiments were carried out four times.

Regulatory T cell is an important subset for homeostasis of intestine 31. We observed the distributions of regulatory T cells in the colons and spleens of mice co‐treated by DSS and wogonin. We avoided non‐specific staining of Foxp3 by measuring the colonic regulatory T cells by flow cytometry gated on CD4^+^ CD25^+^ CD127^−^ cells 32. Flow cytometric analysis of CD4^+^ CD25^+^ CD127^−^ cells of colons was shown in Figure 2A. The absolute number and frequency of CD4^+^ CD25^+^ CD127^−^ cell in total CD4^+^ cell was significantly decreased in mice co‐treated by DSS and wogonin (100 mg/kg), contrary to those of treatments of DSS/solvent or DSS/wogonin (50 mg/kg) (Fig. 2A). The frequencies of CD4^+^ CD25^+^ Foxp3^+^ (Fig. 2B) and CD4^+^ CD25^+^ CD127^−^ cells (Fig. 2C) in the spleens from mice with DSS‐induced colitis were significantly down‐regulated by wogonin (100 mg/kg) treatment, compared with those in the solvent and DSS/solvent groups.

### Wogonin synergizes with IL‐2 to stimulate CD4^+^ T and CD8^+^ T cells *ex vivo*

**Figure 3:**
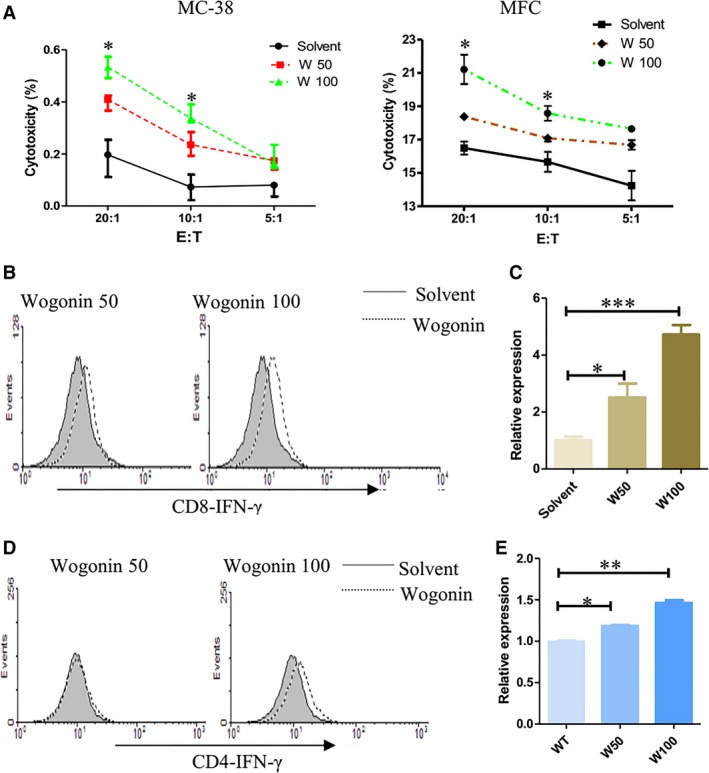
*Ex vivo* stimulations of CD4^+^ and CD8^+^ T cells by wogonin with IL‐2. Splenic CD8^+^ T cells were isolated by flow cytometry and cultured by wogonin and IL‐2 (20 U/ml) stimulations. Lymphocytes were then mixed with murine MFC or MC‐38 cells at indicated ratios. **(A)** Cytotoxicity was measured by an LDH releasing assay. (**B**) Intracellular IFN‐γ production of CD8^+^ T or (**D)** CD4^+^ T cells was also detected by flow cytometry. (**C**) IFN‐γ transcription of CD8^+^ T or (**E)** CD4^+^ T cells detected by real‐time PCR. *, *P* < 0.05; **, *P* < 0.01; and ***, *P* < 0.001. The experiments were repeated thrice.

We observed the effects of wogonin on the biologic activities of CD4^+^ and CD8^+^ T cells in the presence of IL‐2. Biologic function of CD8^+^ T cell is evaluated by its cytotoxicity against target cells and IFN‐γ production. Splenic CD8^+^ T cells in C57BL/6 mice were co‐cultured with MFC gastric cancer cells and MC‐38 colon cancer cells respectively. The cytotoxicity of CD8^+^ T cells was detected by the lactate dehydrogenase release assay. Compared with the solvent, wogonin treatments at 50 or 100 μg/ml concentration significantly enhanced the cytotoxicity of CD8^+^ T cells (Fig. 3A). Wogonin treatments at both dosages also promoted IFN‐γ secretion of CD8^+^ T cells (Fig. 3B) and IFN‐ɣ transcription (Fig. 3C) detected by real‐time PCR. Treatment with 100 μg/ml wogonin up‐regulated IFN‐γ secretion, although 50 μg/ml wogonin showed no significant stimulatory effect on IFN‐γ production by CD4^+^ T cells (Fig. 3D). However, wogonin treatments at both dosages enhanced IFN‐γ transcription (Fig. 3E). Therefore, wogonin synergized with IL‐2 to stimulate functions of CD4^+^ and CD8^+^ T cells *ex vivo*.

### Wogonin inhibits the induction of regulatory T cells *in vitro*

**Figure 4:**
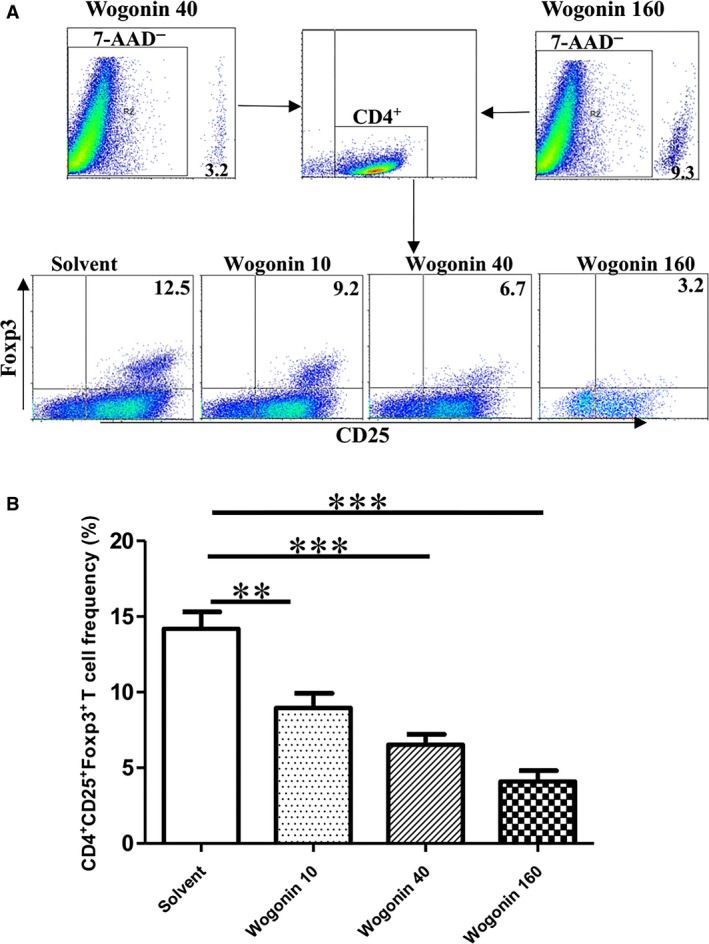
Inhibition of CD4^+^ CD25^+^ Foxp3^+^ cell induction by wogonin treatments *in vitro*. CD4^+^ CD25^−^ T cells were sorted out and cultured in anti‐CD3 antibody pre‐coated wells and stimulated with various concentrations of wogonin and medium containing anti‐CD28 antibody (2 μg/ml), IL‐2 (20 U/ml), and TGF‐β (5 ng/ml) for 5 days. Cells were collected to measure the frequency of CD4^+^ CD25^+^ Foxp3^+^ cells. **, *P* < 0.01; and ***, *P* < 0.001. The experiments were performed thrice.

Splenic CD4^+^ CD25^−^ T cells of normal mice were sorted using a double‐negative MACS to observe whether wogonin could directly inhibit differentiation of Treg cells *ex vivo*. Sorted CD4^+^ CD25^−^ cells with 90% purity were cultured in the presence of IL‐2, CD28 and TGF‐β to induce CD4^+^ CD25^+^ Foxp3^+^ cells. The induction of CD4^+^ CD25^+^Foxp3^+^ T was significantly suppressed by treatments of wogonin at 10, 40, and 160 μg/ml (Fig. 4). Thus, wogonin inhibited the induction of CD4^+^ CD25^+^Foxp3^+^ T cell dose‐dependently.

### Wogonin activates NF‐κB and Erk but down‐regulates STAT3 phosphorylation of CD4^+^ T cells

**Figure 5:**
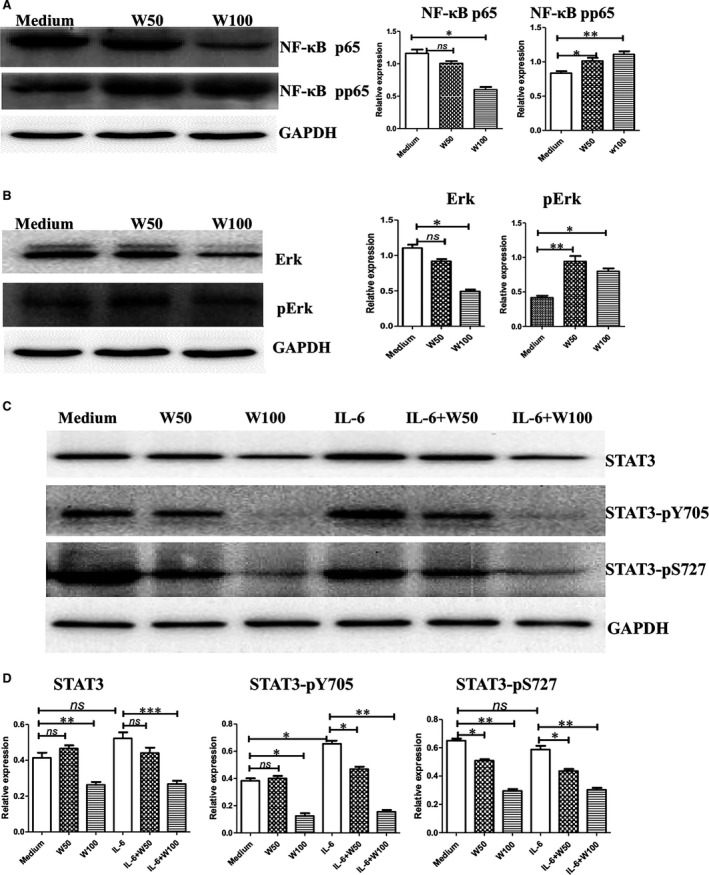
Activation of NF‐κB, Erk, and STAT3 of CD4^+^ T cells by wogonin stimulations. CD4^+^ cells were sorted out by a magnetic cell sorting assay and cultured with IL‐2 (20 U/ml) and various wogonin dosages for 24 hrs. Then, the cells were collected and lysed. (**A**) Phosphorylated NF‐κB, (**B**) Erk, and **(C)** STAT3 were stained with their corresponding antibodies by Western blot. **(D)** Statistical analysis of STAT3 and its activation by wogonin treatments. *, *P* < 0.05; and **, *P* < 0.01. All experiments were performed three times.

Then, we observed the activation of NF‐κB and Erk in CD4^+^ T cells of spleens from normal mice after wogonin treatment. Phosphorylated NF‐κB p65 was enhanced with the stimulation by wogonin (50 or 100 μg/ml), although total p65 was decreased by 100 μg/ml wogonin (Fig. 5A). Similarly, phosphoryl p44/42 (Erk1/2) was activated by wogonin (50 or 100 μg/ml) treatment, although the total p44/42 protein was decreased by 100 μg/ml wogonin treatment (Fig. 5B). The total STAT3 and phosphorylated STAT3‐Y705 and STAT3‐S727 were decreased dose‐dependently after wogonin treatment. IL‐6 can stimulate STAT3 activation in epithelial intestinal cells 33. IL‐6 weakly enhanced STAT3‐Y705 activation, but had no effects of total STAT3 and STAT3‐S727 in CD4^+^ T cells. By wogonin and IL‐6 co‐treatment, CD4^+^ T cells also significantly down‐regulated the activation of total STAT3 and phosphorylated forms (Fig. 5C, D). Thus, wogonin stimulated CD4^+^ T cells by activating NF‐κB and Erk, but down‐regulated the STAT3 signalling.

### Wogonin down‐regulates STAT3‐Y705 and Erk, but activates STAT3‐S727 and NF‐κB of Treg cells

**Figure 6:**
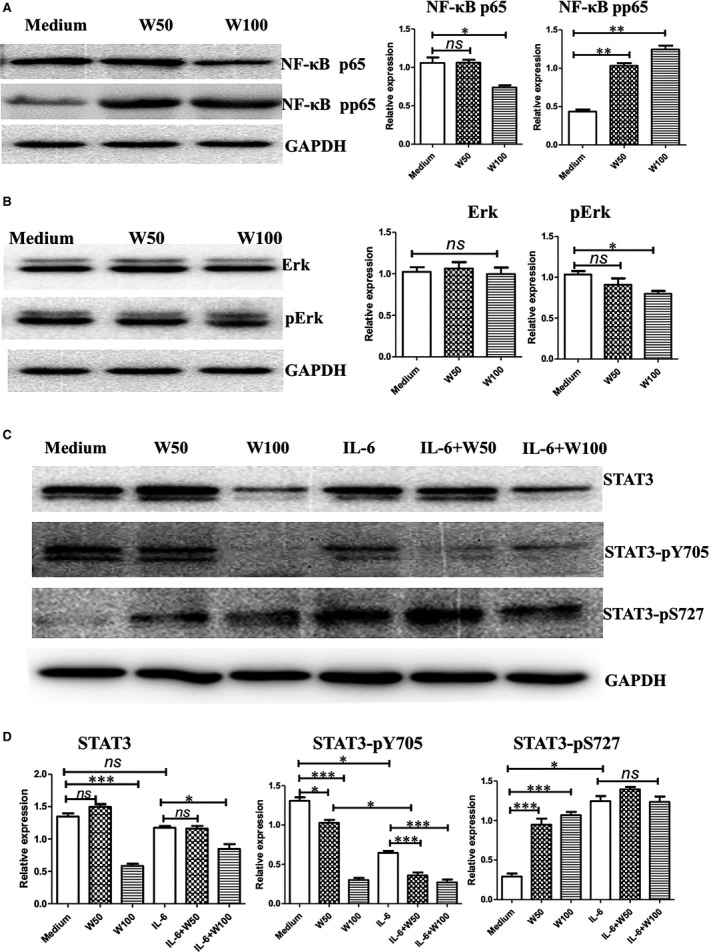
Phosphorylation of NF‐κB, Erk, and STAT3 of regulatory T cells by wogonin stimulations. The CD4^+^ CD25^−^ cells were sorted out and differentiated into CD4^+^ CD25^+^ Foxp3^+^ cells in the presence of IL‐2 (20 U/ml), CD28 antibody and TGF‐β *ex vivo*. The regulatory T cells were cultured with various wogonin dosages for 24 hrs. Then, the cells were collected and lysed. (**A**) Phosphorylated NF‐κB, (**B**) Erk, and **(C)** STAT3 were stained with their corresponding antibodies by Western blot. **(D)** Statistical analysis of STAT3 and its activation by wogonin treatments. *, *P* < 0.05; **, *P* < 0.01; and ***, *P* < 0.001. All experiments were performed three times.

We examined the effects of wogonin on the activation of NF‐κB, Erk and STAT3 of Treg cells which were induced by IL‐2, CD28 and TGF‐β, to elucidate the molecular mechanisms of wogonin on the inhibition of Treg cells. The phosphorylated NF‐κB p65 was up‐regulated by wogonin treatment at both dosages (Fig. 6A). Conversely, wogonin (100 μg/ml) inhibited p44/42 (Erk1/2) phosphorylation, and wogonin at 50 and 100 μg/ml had no significant effects on the total p44/42 (Erk1/2) proteins (Fig. 6B). Wogonin also significantly down‐regulated total STAT3 and the phosphorylation of STAT3 on Tyr705 dose‐dependently, but promoted the phosphorylation of STAT3 on Ser724 of Treg cells. IL‐6 had no effects of total STAT3, but inhibited STAT3‐Y705 activation and increased STAT3‐S727 activation. Wogonin co‐cultured with IL‐6 also significantly down‐regulated STAT3‐Y705 activation, but had no effects on the activation of STAT3‐S727 (Fig. 6C, D).

## Discussion

The anti‐tumour and anti‐inflammatory activities of wogonin have been widely demonstrated. We showed in this study that treatment with relatively high‐dose wogonin (100 mg/kg) could stimulate the function of effector CD4^+^ T and CD8^+^ T cells to exacerbate symptoms of DSS‐induced acute colitis in mice. Moreover, conventional CD4^+^ CD25^+^ Foxp3^+^ regulatory T cells were down‐regulated in mice with colitis. High‐dose wogonin treatment enhanced the biologic activities of CD4^+^ and CD8^+^ T cells by activating NF‐κB and Erk and down‐regulating STAT3‐Y705 activation. Meanwhile, the flavone directly down‐regulated the induction of regulatory T cells *ex vivo* by down‐regulating STAT3‐Y705 and Erk and activating NF‐κB and STAT3‐S727. The study provided evidence that wogonin exhibits distinct effects on different subsets of T lymphocyte, and high‐dose wogonin promotes inflammation.

Wogonin can promote IgA secretion, but down‐regulate IgE levels of mesenteric lymph nodes from mice with DSS‐induced colitis, which were orally administered with 20 mg/kg wogonin for 2 weeks 23. In our study DSS‐mice administrated by wogonin 20 mg/kg *via* peritoneal injection did not have protective effects. Oral drug is easier to induce tolerance 34. The intraperitoneally injected wogonin is absorbed by gastrointestinal tissue and peritoneum, then blood concentration is increased rapidly. Thus, intraperitoneal injection of wogonin is more susceptible to induce inflammation. As a result of intestinal inflammation pre‐induced by DSS, some inflammatory cytokines (eg, IL‐2) will be released. Thus, T cells could be synergistically activated by IL‐2 and wogonin to promote local inflammation.

Wogonin modulates the balance between Th1 and Th2 lymphocytes. This compound enhanced IFN‐γ and IL‐2 production of T cells by co‐treatment with concanavalin A 23. Activation‐induced IL‐4, IL‐5 and IL‐10 secretions were lower in wogonin‐fed mice compared with control mice with DSS‐induced colitis 23. In addition, relative low dose of wogonin (50 μM) suppressed IL‐4 production of murine splenocytes *in vitro* and down‐regulated OVA‐induced Th2 immune responses, particularly IgE and IL‐5 secretion *in vivo* 24. Although Th2 cell‐associated cytokines were not observed, our results confirmed that relatively high‐dose wogonin treatment (50–100 μg/ml equivalent to 176–352 μM) promoted IFN‐ɣ production of CD4^+^ and CD8^+^ cells in the presence of IL‐2 *ex vivo*.

Wogonoside, as the glucuronide metabolite of wogonin, at dosages of 12.5, 25 or 50 mg/kg intragastrically, protects against DSS‐induced experimental colitis in mice by inhibiting NF‐κB and NLRP3 inflammasome activation 35. Firstly, whether the effect of wogonoside is different from that of wogonin need further study. Next high dose of wogonin (50 or 100 mg/kg) were used intraperitoneally and mice were killed on day 8 in our study, which is different in colitis induction and wogonin treatment. In addition, infiltration of activated CD4^+^ and CD8^+^ T cells of colons was only enhanced in mice by 100 mg/kg wogonin treatment. Thus, wogonin may only play an anti‐inflammatory activity in mice with DSS‐induced colitis if administered orally below 50 mg/kg, but exacerbates DSS‐induced colitis by activating T lymphocytes when administered intraperitoneally with the dosage more than 50 mg/kg.

Wogonin (<50 μM) mediates activities against inflammation by down‐regulating the NF‐κB pathway of macrophages and epithelial cells. We demonstrated that wogonin (176–352 μM) could stimulate effector T cell function by activating NF‐κB p65 and Erk p44/42. The cell type, enhanced dose of wogonin and IL‐2 co‐stimulation would contribute to NF‐κB p65 and Erk activation of T cells. The cytotoxicity and IFN‐γ production of effector T cells were stimulated by wogonin in the presence of IL‐2 (20 U/ml). Wogonin alone may have slight stimulatory effects on T cell function but synergize with IL‐2 or other non‐specific stimulators, such as concanavalin A 23, to promote activities of T cells. Wogonin (60 μM) also inhibits Erk p44/42 activation induced by TGF‐β1 of Treg cells 25, which is consistent with decreased Erk activation of Treg cells by wogonin treatment (Fig. 5B).

Wogonin down‐regulated the activation of STAT3‐Y705 in gastric cancer cells (SGC‐7901) at dosages of 10, 40, 160, and 200 μg/ml but showed no significant effects on STAT3‐S727 activation 26. The phosphorylated tyrosine of STAT3 on position 705 was associated with TGF‐β production and expression of B7H1 and MICA of tumour cells 36, 37. Wogonin (50 and 100 μg/mL) also inhibited the activation of STAT3‐Y705 and STAT3‐S727 of CD4^+^ T cells, and inhibited the activation of STAT3‐Y705 of Treg cells like IL‐6, which indicated that STAT3‐Y705 activation is important for Treg cells. Effects and significances of a variety dose of wogonin on STAT3 activation of effector CD4^+^ T or Treg cells need further study.

In conclusion, relatively high‐dose wogonin treatment (100 mg/kg) promoted the onset and severity of DSS‐induced colitis by stimulating the effector CD4^+^ and CD8^+^ cells and reducing the induction of regulatory T cells. Signalling pathways of NF‐κB, Erk and STAT3 were all involved with wogonin‐induced T cell activation. The study confirmed that high‐dose wogonin directly mediated immune‐enhancing function. Consequently, wogonin should be kept in the most appropriate concentration of serum to treat patients with cancer or inflammation.

## Conflicts of interest

All authors have declared no financial conflicts of interest with regard to this work.

## Supporting information


**Figure S1** Effects of high‐dose wogonin on normal mice.
