# Supplementary material for: High‐dose wogonin exacerbates DSS‐induced colitis by up‐regulating effector T cell function and inhibiting Treg cell
Source: J Cell Mol Med. 2016 Sep 19;21(2):286–98. doi: 10.1111/jcmm.12964 (PMC5264153; doi:10.1111/jcmm.12964)
Supplement: Supplementary file 1 — Figure S1 Effects of high‐dose wogonin on normal mice. [file JCMM-21-286-s001.docx]

A


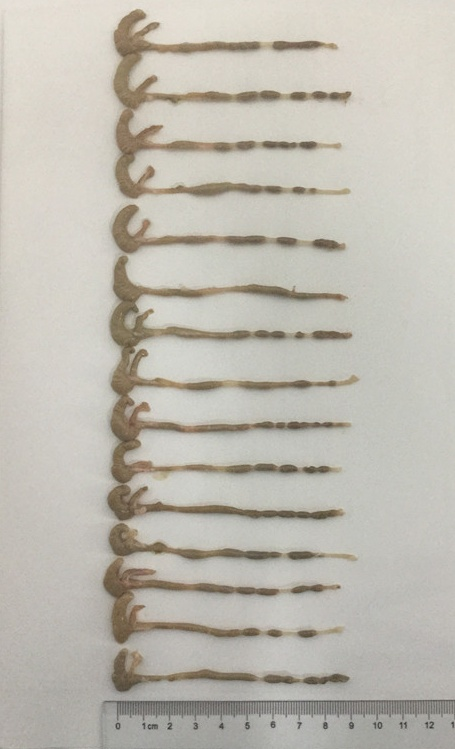


**Solvent**

**Solvent+W50**

**Solvent+W100**

B

C

Solvent+W100 Solvent+W50 Solvent

D


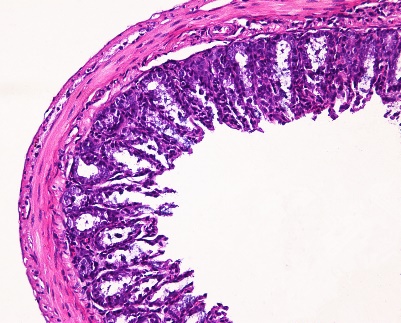

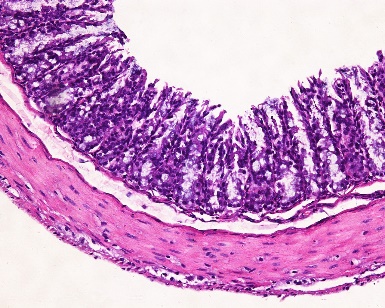

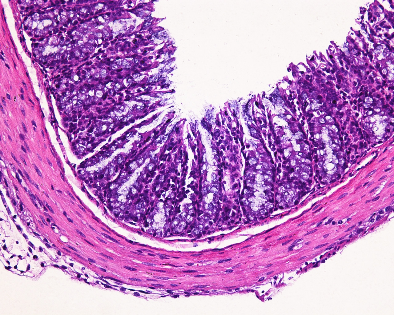


**Supplementary Figure**. Effects of high-dose wogonin on normal mice. (A) Variations of body weight treated by solvent, or wogonin (50 or 100 mg/kg) from day 1 to day 8. (B) Colon length of mice of various treatments. (C) Statistical analysis of variations of colon length. (D) Histological analysis of colon tissue sections by H & E staining (×200 folds).
